# Humming (Simple Bhramari Pranayama) as a Stress Buster: A Holter-Based Study to Analyze Heart Rate Variability (HRV) Parameters During Bhramari, Physical Activity, Emotional Stress, and Sleep

**DOI:** 10.7759/cureus.37527

**Published:** 2023-04-13

**Authors:** Gunjan Trivedi, Kamal Sharma, Banshi Saboo, Soundappan Kathirvel, Ashwati Konat, Vatsal Zapadia, Poojan J Prajapati, Urva Benani, Kahan Patel, Suchi Shah

**Affiliations:** 1 Society for Energy & Emotions, Wellness Space, Ahmedabad, IND; 2 Cardiology, Dr. Kamal Sharma Cardiology Clinic, Ahmedabad, IND; 3 Department of Endocrinology, Diabetes Care & Hormone Clinic, Ahmedabad, IND; 4 Community Medicine, Postgraduate Institute of Medical Education and Research, Chandigarh, IND; 5 Department of Zoology, Biomedical Technology and Human Genetics, Gujarat University, Ahmedabad, IND; 6 Internal Medicine, B J Medical College, Ahmedabad, IND; 7 Smt. NHL Municipal Medical College, Internal Medicine, Ahmedabad, IND; 8 Internal Medicine, AMC MET Medical College, Ahmedabad, IND

**Keywords:** lifestyle, bhramari pranayama, humming, heart rate variability, stress

## Abstract

Objective

In this study, our goal was to understand the comparative impact of humming, physical activity, emotional stress, and sleep on several heart rate variability (HRV) parameters, including the stress index (SI), and to assess the effectiveness of humming (simple Bhramari) as a stress buster based on the HRV parameters.

Methods

This pilot study assessed the long-term HRV parameters of 23 participants in terms of four activities: humming (simple Bhramari), physical activity, emotional stress, and sleep. The single-channel Holter device measured the readings, and data was analyzed using Kubios HRV Premium software for time and frequency-domain HRV parameters, including the stress index. Regarding statistical analysis, single-factor ANOVA followed by paired t-test was used to compare the results of HRV parameters "during" the four activities to understand if humming generates the outcome to enhance the autonomic nervous system.

Results

Our findings revealed that humming generates the lowest stress index compared to all three other activities (physical activity, emotional stress, and sleep). Several additional HRV parameters also supported the positive impact on the autonomic nervous, equivalent to stress reduction.

Conclusions

Humming (simple Bhramari) can be an effective stress-buster based on the assessment of several HRV parameters during its practice and in comparison with other activities. A regular daily humming routine can help enhance the parasympathetic nervous system and slow down sympathetic activation.

## Introduction

Stress is known to influence autonomic function, disease progression, and related quality of life in several chronic diseases. Specifically, (a) acute and chronic stress significantly increases the risk of heart disease and increases the mortality risk for heart disease subjects [1]; (b) evidence confirms that emotionally stressful experience is associated with endocrine disorders such as diabetes mellitus [2], and stress can play a role in the onset of diabetes and adversely impact glucose levels and quality of life; (c) stress is also linked to lung disease, e.g., compared to healthy individuals, stress is associated with a greater level of depression in individuals and poor quality of life for those with chronic obstructive pulmonary disease (COPD) or asthma [3]; finally (d) epidemiological and clinical studies over the past 30 years have provided strong evidence of the links between chronic stress, depression, social isolation, and cancer progression [4].

Poor lifestyle choices such as sleep disruption, increased work-life balance challenges, reduced physical activity, and calorie-heavy diet, along with emotional stress, impact the quality of life, decrease heart rate variability (HRV) [beat-to-beat variations in heart rate (HR)], and lead to increased prevalence of metabolic syndrome (set of measures defining the risk for chronic disease) and eventually chronic disease [5]. The link between stress and the autonomic nervous system and the role of HRV is well-documented. Evidence has shown that relaxation, slow breathing, or any stress reduction intervention increases HRV [6]. Hence, there is an opportunity to integrate an activity that can reduce stress, enhance the autonomic system (including HRV), and improve sleep quality into a lifestyle for stress management.

Humming (simple Bhramari) is a simple activity with several positive benefits, such as a decrease in HR, an increase in HRV, an enhancement of autonomic and lung function, and an increase in attention and sleep quality [7]. This pilot study aimed to understand the uniqueness of HRV parameters during humming compared to several other activities to validate the idea further. Beyond the statistical comparison, the idea behind the pilot was to learn how consistently this uniqueness manifests between such activities and its consistency across several individuals. Given the complexities and relative ambiguities involved in the HRV parameters, the study also highlighted some of the parameters' limitations and explored future research ideas and potential. The insights from such a pilot study can provide more validation and create a context in which the humming (simple Bhramari) could be evaluated as a lifestyle intervention - especially as a stress buster - compared to several other activities performed during the day and the night. Several studies have measured the HRV of different individuals [8], but not many studies have explored how HRV changes during various activities [9]. An essential element that the study explored is the ability of HRV biofeedback-related humming practice to significantly increase the HRV parameters, something that does not happen during stress or physical activity. It is also possible that humming creates a unique pattern of the heart's signal (lower sympathetic activation, higher parasympathetic tone), which the study investigated

The importance of activities for increasing the HRV

Reviews conducted earlier have highlighted the broad-based potential for choosing a lifestyle to increase HRV and decrease HR (and thereby influence the reduction in resting HR). In other words, the goal is to incorporate a practice that could achieve increased HRV (which includes reduced HR) through regular practice, as illustrated in several HRV biofeedback and yoga reviews [10-12]. There are several core principles behind the outcome (increased HRV) as per the above evidence, as highlighted below:

The ability to influence the cardiorespiratory resonance during the practice enhances heart and lung functions and optimizes energy expenditure. The practice increases autonomic tone and baroreflex sensitivity [7].

Even after the training is completed, the practice increases breathing quality and consistency through increased HRV, lung function, etc. The enhancements continue to help even when the individual is consciously not doing the activity [13].

Stress reduction is followed by the enhancement of autonomic function. Reducing stress is one of the critical enablers for increased cognition. Recent research highlighting the area of cerebrospinal fluid (CSF) flow further supports this claim [14,15]. The use of sound vibration further enhances cognition, focus, and attention [16].

Beyond the above points, the additional possibilities may include increased air exchange in the lung during simple Bhramari due to increased nitric oxide levels during the humming process [7,17-19]. Several studies have highlighted that HRV parameters improve during meditation [20,21]. Similar changes are found after prolonged humming (Bhramari) and HRV biofeedback practices (discussed earlier) [12]. Only one study has evaluated changes during Bhramari practice so far to the best of our knowledge.

This pilot provides new insights into how a structured, simple Bhramari practice stacks up against various other activities during the 24-hour duration for stress management based on the hypothesis that simple Bhramari practice generates better HRV parameters than several different activities regarding emotional stress, physical activity, or even sleep. The HRV parameters chosen were stress index (SI), the standard deviation of normal-to-normal Intervals (SDNN), low frequency (LF)/high frequency (HF) ratio, and total power. The stress index (based on Baevskey’s formula that uses the 50 milliseconds histogram window representation of the RR interval) was also added as a core HRV parameter for a better understanding of stress levels for the chosen set of activities [22,23,24]. The equation for the stress index shown below was calculated by the Kubios HRV Premium software.

Stress Index (SI) = (AMo*100%) / (2Mo*MxDMn)

In the equation, Mo is the median of the RR interval in seconds. MxDMn is the width of the histogram showing the degree of variability in RR intervals. AMo is the height of the normalized RR interval histogram (bin width of 50 ms).

With a specific focus on stress, two activities - emotional stress and physical activity - were added along with sleep to explore the differences and similarities in HRV parameters for these activities with a specific focus on humming as a stress buster.

## Materials and methods

Study design

This pilot cross-sectional study (analytical) was designed to examine the long-term HRV parameters in terms of four activities: humming (simple Bhramari), physical activity, emotional stress, and sleep. The protocol was approved by the institutional ethics committee (ref. no: ECR/274/Inst/GJ/2013/RR-19, dated 27/10/2020), and it was registered with the Clinical Trials Registry, India (registration no: CTRI/2020/12/030131). Informed consent was obtained from all participants before their enrolment in the study.

Study participants

A total of 29 participants were recruited based on word-of-mouth and social media-based requests with a determined sample size of 20 after considering dropouts and incomplete participation (based on activity assessment). Participants included both men and women aged between 18 and 60 years who are functional individuals with normal health or with chronic disease (e.g., diabetes, hypertension, pulmonary disease, etc., and who walk regularly). Participants with pre-existing illnesses that can interfere with HRV viz. recent acute coronary syndrome in the last three months, high grade of heart blocks, patients with pacemaker implantation or those scheduled for implantation, or those with any history of stroke or primary or secondary dysautonomia including diabetic neuropathy were excluded.

Study procedure

**Figure 1:**
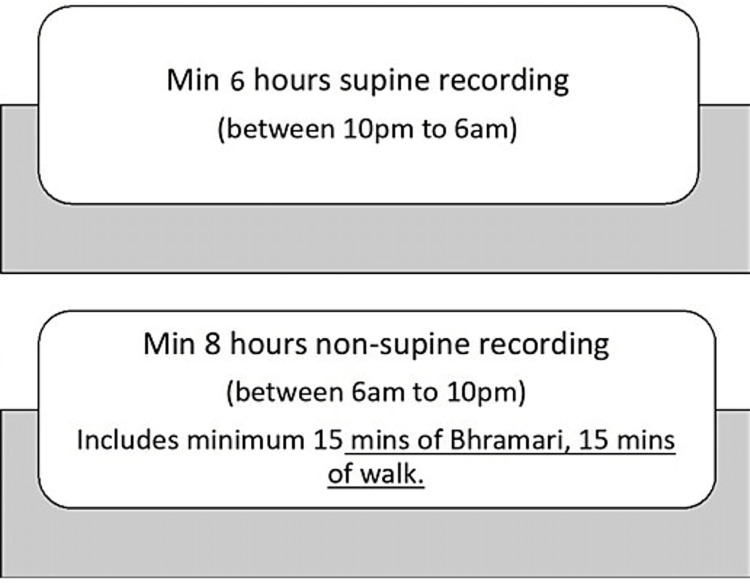
Process for Holter recording

Each participant agreed to wear the single-channel Holter device (Bittium Faros 180; Bittium Corporation, Oulu, Finland) for at least 16 hours, including a minimum of eight awake hours and six hours during sleep (supine recording at night). During this time, the individual highlighted the activities such as a physical activity that involved movement (such as walking, jogging, or bicycling), sleep (at night), simple Bhramari (at least 15 minutes), and any stressful activity (this was determined by the combination of HRV parameters and validation from the individual as captured and noted in the activity sheet, again for at least 15 minutes). The process for Holter recording is presented in Figure 1 and activities are detailed in the next section.

Activities for analysis

The activities are described in detail below:

(a) Humming or Simple Bhramari - this practice involves Bhramari Pranayama without using any specific hand gestures (mudras). Participants were requested to inhale for up to three to four seconds followed by an exhalation of about six to eight seconds while making the humming bee sound or Bhramari Pranayama sound. The flexibility encouraged participants to adjust their inhalation and exhalation length in a way that allowed them to continue the practice for a longer time. (b) Physical activity that included movement (including walking, jogging, bicycling, or sports). (c) Stress (mental or emotional stress), where the participant was requested to highlight the beginning and end time of any stressful situation such as being stuck in a traffic jam or a difficult phone call, or an emotionally intense event. (d) Sleep - this was captured as the start and end time for sleep during nighttime when the participant was supine. Afternoon naps were not included as part of the study. Each participant was trained using an HRV biofeedback device to practice humming based on the practice instructions highlighted above. Guidelines (duration, specifications) of each activity were also reviewed with each participant in detail to ensure compliance. As described in the next section, after the observational readings were completed, based on the participants’ notes, each activity's “start” and “end” time and the parameters were validated by the subject matter expert to ensure compliance with the actual results.

From a total of 29 participants who joined the study, 23 completed the necessary recordings and made relevant notes for each of the four activities highlighted above. 

Assessments and analysis

Bittium Faros 180, a single-channel ECG device, was used to measure the HRV signals from each participant. The device sampling rate for Bittium Faros selected was 500 Hz, which is far above the recommended minimum of 250 Hz (several studies have validated this) [25,26].

The participants made notes of the activities and returned the sheet. The data from the device (.EDF file) was imported for further analysis into Kubios Premium HRV software. The output of the HRV data was imported into a Microsoft Excel sheet. Each activity was marked based on the participant's notes by an independent individual who validated the activity with the subject to ensure the accuracy of the validation. In other words, the marking of various actions and duration was done based on the validation between the participant's notes and actual HRV data. Kubios Premium HRV software analysis included automatic error correction and removal of any errors (usually at the start or end of the recording or any in-between time duration, such as errors appearing because of the device getting disconnected or removed from the chest). The data analysis was performed using Microsoft Excel statistical features on a Windows PC.

## Results

A total of 23 individuals completed the study, including 13 men and 10 women. All 23 participants (including two individuals with type 2 diabetes and two individuals with primary hypertension) completed humming (simple Bhramari), physical activity, emotional stress, and sleep. The average age of the participants was 38.83 ±14.55 years. The summary of essential HRV parameters (average and standard deviation) is presented below in Table 1.

**Table 1:** Heart rate variability parameters during humming, physical activity, sleep, and stress *Indicates the statistical significance of the specific parameter and activity combination vs. other activities, as calculated using single-factor ANOVA followed by the paired t-test, p<0.05 RMSSD: the root mean square of successive differences between normal heartbeats; SDNN: standard deviation of normal-to-normal Interval: LF: low frequency; HF: high frequency: ANOVA: analysis of variance

Activity	Stress index (SI)	Heart rate	RMSSD	SDNN	Total power	LF/HF ratio
Humming	9.26 ±2.49*	82.54 ±7.92	41.40 ±29.37	61.83 ±24.68*	4318.82 ±3350.67*	13.52 ±19.17*
Physical activity	23.36 ±13.19	103.27 ±12.21	18.66 ±21.01	26.73 ±21.68	1003.84 ±1700.29	5.24 ±2.58
Sleep	10.94 ±3.47	70.35 ±7.19*	45.62 ±28.83	47.76 ±20.82	2750.97 ±2538.47	2.26 ±1.47
Stress	21.68 ±7.39	97.21 ±14.52	14.81 ±10.19	22.41 ±12.08	648.43 ±891.78	5.70 ±3.42

There are several interesting insights gained from the results. The single-factor ANOVA confirmed a unique difference in specific parameters for each activity shown in the table above. The paired t-test followed the ANOVA to understand the uniqueness of each parameter (i.e., stress index, heart rate, RMSSD, SDNN, total power, and LF/HF ratio) between the activities. The insights from the paired t-test to further understand the details are highlighted below.

1. Stress index (SI) was statistically significant for humming as compared even to sleep and, of course, the other two activities (p<0.05). In other words, the stress index was significantly lower during humming than during sleep, physical activity, and, emotional stress (p<0.00073, 0.0000, and 0.000, respectively). The stress index was also (expectedly) lower during sleep than during physical activity and emotional stress. However, there was no statistically significant difference in stress index between physical activity and emotional stress (p=0.29). The statistical data confirms that humming and sleep help reduce stress (though humming generates a lower stress index than sleep). 

2. The heart rate between all the activities was different, and this significant statistical difference was confirmed based on the paired t-test data. However, in absolute value, the heart rate was the lowest during sleep (lower than humming, p<0.05). At the same time, during the activities when the person was awake (physical activity, stress, and humming), the humming resulted in the least heart rate (statistically significant p<0.001 for both activities compared to humming). 

3. SDNN, measuring the standard deviation of normal-to-normal heart signal intervals, was statistically significant for humming compared to all three activities, with a p-value of <0.05 (with sleep, physical activity, and stress). However, there was no statistically significant difference in SDNN between physical activity and emotional stress (p=0.071, >0.05). Hence, the SDNN value is also unique during humming compared to all other activities, confirming the ability of humming to generate unique oscillations of the heart's signals. A similar pattern of statistical significance was also observed for total power during humming. To summarize, humming generates better SDNN and total power (statistically significant) even compared to sleep.

4. RMSSD value was higher for humming than for physical activity and emotional stress (statistically significant with p-values of 0.033 and 0.001, both <0.05). However, RMSSD was higher for sleep than for humming, though the difference between the two activities was not statistically significant (p=0.28, i.e., >0.05). From a statistical perspective, we can conclude that humming significantly increases RMSSD compared to physical activity and emotional stress. However, sleep generates a higher RMSSD value, but the increase is not statistically significant, as captured earlier.

5. LF/HF ratio was the highest during humming, consistent with the finding that the total power is distributed primarily in the LF band region. This increase is statistically significant compared to sleep and physical activity but not stress (p=0.061, i.e., >0.05). At the same time, the comparison of LF/HF between stress and physical activity indicated that the increase during stress is not statistically significant compared to physical activity (p=0.45, i.e., >0.05).

Traditionally the LF/HF power ratio (LF/HF ratio), especially during long-term recording (~24 hours), provided the sympathetic nervous system (SNS) activity (denoted by LF power) divided by parasympathetic nervous system (PNS) activity (represented by HF power). Hence, a higher LF/HF ratio could indicate an increased sympathetic tone or fight-or-flight mode. In contrast, a lower LF/HF ratio could denote parasympathetic tone or rest and digest mode [27]. This understanding has been questioned recently, mainly due to the cut-off values of various frequency bands. Hence, the traditional interpretation is not relevant despite this study's long-term recording [28]. Humming done at slow breathing with consistent breathing length (similar to HRV biofeedback) could increase respiratory sinus arrhythmia. Hence, the LF band power is significantly increased, and the power in the HF band decreases, raising the LF/HF ratio significantly. Therefore, a higher LF/HF ratio is a significant finding indicating very good total power concentrated mainly in the LF band. A higher LF/HF ratio shows increased oscillations of the heart at a frequency that falls in the LF band. The normalized power in humming shifts to LF bands (the maximum compared to all other activities). (LF nu power during humming across all the participants averaged 80.9 compared to 45.5 during sleep. LF nu power for stress and physical activity was 22.7). At the same time, the total power is maximum during that activity compared to all other activities. This confirms that the total power increases and the shift in the LF band are based on the categorization that has been decided for various bands.

The humming activities consistently generated a statistically significant improvement in stress index, SDNN, total power, and LF/HF ratio compared to all other three activities. Moreover, the increase in RMSSD and reduction in HR during humming was statistically significant compared to emotional stress and physical activity but not with sleep. There are several insights gained from the above results, which are discussed in the next section. Key highlights are shown in the visual below, highlighting the stress-reducing and HRV-enhancing benefits of humming compared to all three activities.

## Discussion

To the authors' knowledge, this is a unique pilot study comparing specific time- and frequency-domain HRV parameters for a stress-reduction activity such as humming with physical activity, emotional stress, and sleep. Several studies have explored machine learning and artificial intelligence in recognizing activities based on the HRV parameters [29,30]. Most of the studies have used core RR interval, ECG, and accelerometer data, resulting in reasonably good accuracy in assessing various activities. This study took a different approach by focusing on how simple Bhramari creates unique HRV parameters through cardiorespiratory coupling and comparing these parameters with other activities. Several aspects of the results are discussed in this section.

First, while the stress index has been used in many studies to understand the impact of the activity on the nervous system [31], its use in comparing stress (both physical activities and stress) with stress-reducing activities (both sleep and humming) is one unique aspect of this pilot. The results confirm that either humming is statistically significantly better compared to both stressors or even sleep (SDNN, total power, LF/HF ratio, and stress index) or it provides outcomes similar to sleep (i.e., stress reduction activity) compared to stress (Both RMSSD and heart rate during humming are statistically significantly higher compared to both stressors but not better compared to sleep in terms of its positive impact on the autonomic nervous system). 

Second, the enhanced HRV parameters validate and provide further possibilities for research in a more structured manner. Increased SDNN, increased total power, decreased stress index, and high RMSSD (compared to physical activity and stress but lower than sleep) provide good validation that humming indeed creates a unique physiological and psychological state that could influence autonomic balance. Better HRV parameters compared to sleep may indicate that the "humming" state is unique and can sustain respiratory components through a resonance frequency even lower than 0.1 Hz. The use of sound vibrations at the lower frequency may have a potentially influencing role in CSF flow that could be studied further [14,32]. The lower stress index and SDNN confirm the unique RR variations during humming. 

Third, the higher RMSSD value during sleep than humming is an exciting finding and needs to be evaluated along with the evidence that RMSSD is influenced more by the vagal tone. SDNN represents the activity of both branches of the autonomic nervous system (ANS) [33]. As indicated by higher SDNN, stress index, and total power values, sympathetic activity reduction could influence the short-term increase in SDNN during "humming". SDNN increase is also associated with increased focus and attention, something that HRV biofeedback literature reviewed earlier has also highlighted. Hence, the unique value-addition of humming, beyond obvious stress reduction, through higher SDNN could be an increased focus while balancing the ANS. There is evidence to suggest that with the regular practice of Bhramari, both SDNN and RMSSD can be increased [13]. A recent study evaluated mindfulness practices' impact on HRV after 10 days of practice [21] and discovered a dose-response relationship between meditation practice and HRV. The benefits extended beyond HRV through improved sleep and reduced stress. Therefore, engaging in a structured but simple Bhramari practice as a daily lifestyle intervention could prove to be potentially beneficial. 

**Figure 2:**
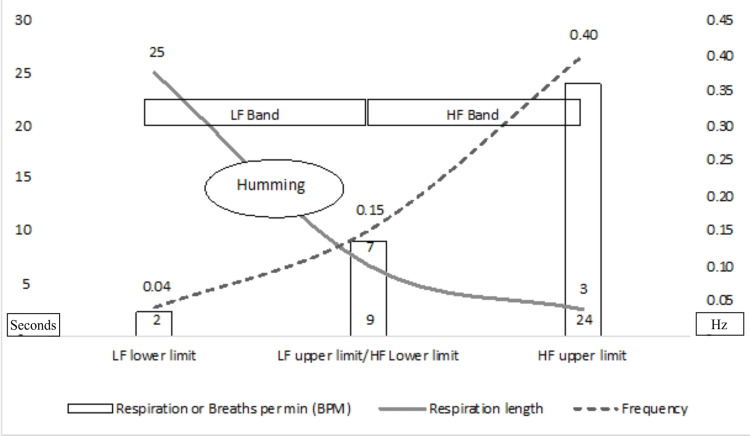
Heart rate variability frequency bands (in Hz), respiration length (in seconds), and frequency (in BPM) LF: low frequency; HF: high frequency

Fourth, the high and statistically significant LF/HF ratio value must be interpreted carefully. As discussed earlier, the heart's frequency signals end up in the LF band during humming. This unique aspect of slow breathing is that the LF frequency range contributes to the increased LF/HF ratio. The total power is statistically significant and maximum during humming, validating the argument that humming results in increased total power. The power is concentrated in the LF band and, therefore, the percentage share of HF power during humming is reduced. The ambiguity involved in interpreting HF power data "during" HRV biofeedback or slow breathing is not new [34]. The pilot results provide a perspective behind the ambiguity, as highlighted in Figure 2. The circle showing the "humming" respiration range around 12 seconds translates to 5 BPM or 0.08 Hz, which happens to fall into the LF band. This results in the power moving in the LF range resulting in very high LF power and a reduction in HF band power. This creates ambiguity since it is believed that in terms of frequencies, the SNS does not appear to produce rhythms above 0.1 Hz, and the PNS impacts the heart rhythms down to 0.05 Hz (or 20 seconds) [27]. In this context, with total power covering the energy in all the frequency bands, it is interesting to note that HF power (nu) is lowest during humming. This difference compared to other activities is statistically significant. This also explains higher LF/HF values even when compared with the number during sleep and this does not mean there is sympathetic activation during humming as validated by several other parameters including total power. Hence, it is appropriate to review the total power data vs. changes within the band that provides ambiguity due to resonant frequency-related mingling within the LF band. Research has highlighted that total power is significantly higher during sleep and lying down (reading) activities than in sitting, reading, or other physical activities [35]. With the above interpretation, while the power during humming shifts to the LF band and the HF band power reduces, the total power is still very high. The significant variations in heart rate could contribute to this during the humming activity at around 0.08 Hz. Significantly higher total power during humming is also, in this context, a unique benefit highlighted by this pilot study.

There are several reasons for using Bhramari compared to passive meditation since (a) active meditation is easier for beginners and reduces the impact of adverse reactions such as anxiety or panic attacks, (b) meditation, in general, results in improved HRV and lower HR, and (c) there is demonstrated evidence of how humming (Bhramari) over longer-duration impacts mind-body parameters measured through HRV [13,20].

This pilot provides several important insights despite some of the limitations of the method and HRV measurements (covered in the next section). Most of the parameters (except RMSSD) related to parasympathetic activation and sympathetic deactivation increase during humming with statistical significance compared to sleep. This indicates that the HRV during humming is unique. This voluntary pattern does not happen during other activities, including long-duration sleep or slow breathing (as discussed and demonstrated in earlier experiments). This outcome, in the context of several established protocols of HRV biofeedback benefits [10,36,37], provides a unique benefit that (a) not only does humming provide a new insight that the resonance frequency does not need to be 0.1 Hz, it can be even lower, (b) humming extends the idea of HRV biofeedback and adds more pronounced positive impact on several HRV parameters, and (c) the improvement in HRV parameters is even better than similar parameters during "sleep" also. Hence, these unique HRV patterns, when repeated over several weeks and months, generate significant enhancement in physiology, affective components, cognition, and respiratory and cardiovascular parameters [38].

Traditionally the LF/HF power ratio (LF/HF ratio), especially during long-term recording (~24 hours), provided the SNS activity (denoted by LF power) divided by PNS activity (denoted by HF power). Hence, a higher LF/HF ratio could indicate an increased sympathetic tone or fight-or-flight mode. In contrast, a lower LF/HF ratio could denote parasympathetic tone or rest and digest mode [27].

This assumption that the LF/HF ratio measures sympathovagal (or SNS-PNS) balance is also questioned [29], by arguing that LF power is not a pure index of SNS activity and PNS-SNS interactions are not linear but complex and non-linear. Hence, the LF/HF ratio measurement, especially during short-term recording, depends on the measurement conditions and is not comparable to the 24-hour recordings. A study explored different LF/HF ratio interpretations to address this challenge [39]. However, this interpretation also does not address the challenges during the resonant breathing frequency or humming at a frequency that falls in the LF band since these activities significantly increase LF power compared to HF power. The results of the study (especially the LF/HF ratio), when discussed in this context of ambiguity of the LF/HF ratio, still provide significant clarity about the positive impact of humming on the frequency-domain parameters.

Limitations and future possibilities

Humming provides the unique advantage of generating 0.1 Hz or lower frequency oscillations on the heart's overall functioning and impacts the brain pathway through afferent signaling, very similar to how the HRV biofeedback works [40,41]. This cardiorespiratory resonance state creates and maintains the optimum energy state of the heart's functioning, and hence the humming method could have long-term potential benefits as a lifestyle intervention. Recent findings about the linkage between respiration frequency (especially at or below 0.1 Hz) and CSF flow add unique benefits that hold massive promise while needing further research [14,32]. In this context, this pilot explored if the oscillations (measured through currently established HRV parameters) provide additional insights about HRV "during" humming and how it compares with a few other activities that we usually do during the 24-hour duration. The pilot's objective was not to make a perfect comparison between various activities but to learn and get some early insights that can be integrated into future studies. 

The HRV patterns follow a circadian rhythm and vary by sleep stages, and identifying the HRV for each sleep stage requires complex analysis and artificial intelligence algorithms [39]. Hence, for this study's purpose, the HRV parameters for sleep were represented by "supine sleep" at night. While it is not ideal for comparing longer-duration HRV parameters with shorter-duration patterns, the idea behind taking different measurement duration windows for the pilot was to assess if there is any unique insight that can transcend the limitation of "duration" as well as constraints in HRV parameter evaluations such as LF/HF ratio of frequency band-specific limitations due to respiration component. 

The ability to generate strong LF frequency-dominant power and oscillations sets the humming practice apart from other activities, with the possible exception of slow breathing (within the LF band range). Because of this, the LF/HF ratio during humming is also the "highest" compared to other activities. While the limitations of the LF/HF ratio interpretations are discussed earlier, the highest SDNN, total power, and lowest stress index across all the activities make a strong case for a more detailed study in this area to address the limitations presented by the LF/HF ratio.

Finally, the small sample size in the study is a limitation that can be addressed in future research related to assessing various parameters for any stress-reduction and energy-balancing activity.

Several exciting possibilities can be explored based on the findings of the study. It would be interesting to understand how HRV parameters during humming stack up compared to various sleep stages. Low-frequency respiratory waves during NREM sleep are linked to increased CSF flow. Hence, it would be worthwhile to understand the impact of low-frequency respiration waves and sound vibrations on CSF flow. Future research can also study the long-term impact of at least 20-minute-long daily humming practice on HRV and sleep-related HRV data.

Together, these findings indicate that the patterns created by humming (simple Bhramari) are unique even when observed based on 24-hour measurements and compared with other crucial activities such as stress, physical activity, and sleep. By incorporating such activity for a duration of at least 10-15 minutes twice a day, the individual gains several benefits, including (a) benefits from HRV biofeedback research, (b) benefits of generating low-frequency respiratory oscillations and potential benefits on CSF flow, and (c) enhancement in cardiovascular, respiratory, and psychological parameters.

## Conclusions

This pilot study provides several insights into the uniqueness of HRV signals during humming (simple Bhramari) compared to activities such as (emotional) stress, physical activity, and sleep in several individuals as measured through a Holter device. The benefits related to several enhanced time- and frequency-domain parameters obtained through the voluntary and structured practice of simple Bhramari prompt us to make a case for the activity as an effective lifestyle intervention and as an antidote for stress. The increased HRV can eventually increase focus and attention, enhance the quality of life and cardiovascular and lung parameters, and improve baroreflex function. Future work should address the limitations of the study by comparing data across several activities of similar duration beyond the current scope, by adding more samples (such as additional physical activity and active vs. passive meditation), and by focusing on specific HRV parameters that are less ambiguous and explore how such interventions could enhance the CSF flow, sleep quality, attention, and focus.
